# Evolutionary obstacles and not C–F bond strength make PFAS persistent

**DOI:** 10.1111/1751-7915.14463

**Published:** 2024-04-09

**Authors:** Lawrence P. Wackett

**Affiliations:** ^1^ Department of Biochemistry, Molecular Biology and Biophysics and Biotechnology Institute University of Minnesota St. Paul Minnesota USA

## Abstract

The fate of organic matter in the environment, including anthropogenic chemicals, is largely predicated on the enzymatic capabilities of microorganisms. Microbes readily degrade, and thus recycle, most of the ~100,000 commercial chemicals used in modern society. Per‐ and polyfluorinated compounds (PFAS) are different. Many research papers posit that the general resistance of PFAS to microbial degradation is based in chemistry and that argument relates to the strength of the C–F bond. Here, I advance the opinion that the low biodegradability of PFAS is best formulated as a biological optimization problem, hence evolution. The framing of the problem is important. If it is framed around C–F bond strength, the major effort should focus on finding and engineering new C–F cleaving enzymes. The alternative, and preferred approach suggested here, is to focus on the directed evolution of biological systems containing known C–F cleaving systems. There are now reports of bacteria degrading and/or growing on multiply fluorinated arenes, alkenoic and alkanoic acids. The impediment to more efficient and widespread biodegradation in these systems is biological, not chemical. The rationale for this argument is made in the five sections below that follow the Introduction.

## Graphical abstract

C–F bonds can be cleaved by microbes. However, evolution must overcome many obstacles to biodegrade PFAS.

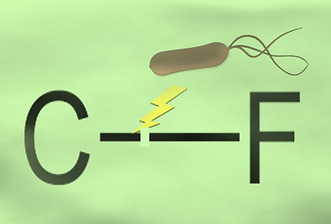

## INTRODUCTION

Solving a problem simply means representing it so as to make the solution transparent. Herbert Simon

Nothing in biology makes sense except in the light of evolution. Theodosius Dobzhansky

The quotes above are relevant to the idea that framing the cause of a problem is the key to its solution and that an evolutionary perspective is important for understanding biological problems. This applies to the environmental biodegradation, or lack thereof, of PFAS. Various regulatory agencies define PFAS differently. Here, I will refer to per‐ and polyfluorinated compounds containing one or more CF_2_‐ or CF_3_‐groups broadly as PFAS (Wang et al., 2021).

The opinion I advance here is that the problem of PFAS persistence is best framed in the context of evolution, not chemistry. The alternative chemistry‐based explanation, advanced by many, is that the C–F bond is sufficiently strong such that biodegradative enzymes are ill‐equipped to achieve C–F cleavage. Contrary to that, there are continuing to emerge examples of microbial and enzymatic defluorination of PFAS and this suggests that enzymes are capable of cleaving C–F bonds on multiply‐fluorinated carbon centers. However, when that occurs, there is little energy to be obtained in oxidising highly fluorinated aliphatic compounds and multiple enzymes need to arise simultaneously to metabolize this non‐natural class of compounds. Compounding that, C–F bond cleavage produces highly toxic fluoride anion, imposing a strong negative selection against PFAS biodegradation. In that context, PFAS persistence is best reframed in terms of evolution and that framing will move us faster to obtaining solutions.

## MANY TYPES OF C–F BONDS IN PFAS ARE NOW KNOWN TO BE CLEAVED ENZYMATICALLY

The failure of many polyfluorinated compounds to undergo biodegradation in multiple environments is often attributed to the strength of carbon‐to‐fluorine (C–F) bonds (Berhanu et al., 2023; Key et al., 1997; Lorpaiboon & Ho, 2023; Xiao et al., 2023). The stability of PFAS to thermal, oxidative, and photochemical degradation is well known (Achiha et al., 2010; Haupt, 2021; Krespan, 1965; O'Hagan, 2008). It is irrefutable that the C–F bond is one of the strongest bonds known. However, it does not necessarily follow that this is the major explanation for PFAS persistence to biodegradation.

For example, it has been suggested that enzymes may react with monofluorinated compounds such as fluoroacetate but compounds with –CF_2_‐ and –CF_3_‐groups will resist enzymatic attack (Alexandrino et al., 2018; Frömel & Knepper, 2010; Johns & Stead, 2000; Sáez et al., 2008). The bacterial defluorination of –CF_3_‐groups appended to aromatic rings has been shown to occur via ring oxidation reactions that produce quinone methide intermediates that are susceptible to complete defluorination to produce a carboxylic acid (Bygd et al., 2022; Kiel & Engesser, 2015). More recently, enzymes have been shown to directly catalyse defluorination of –CF_2_‐groups, specifically α,α‐difluoro carboxylic acids (Khusnutdinova et al., 2023), a common moiety in a significant number of commercial PFAS for which persistence is notorious, and environmental removal is avidly sought (Sznajder‐Katarzyńska et al., 2019). The enzymes are members of the α/β‐hydrolases, a very widespread and highly evolvable enzyme superfamily (Holmquist, 2000). α/β‐Hydrolase enzymes catalyse a very wide array of reactions, such as ester and epoxide hydrolysis, lyase reactions, group transfer, and dehalogenation (Jochens et al., 2011). A sub‐class of α/β‐hydrolase enzymes has been known to catalyse defluorination of monofluoroacetate (Chan et al., 2011), a highly toxic natural product organofluoride compound that is biosynthesised by some plants and microbes (O'Hagan & Deng, 2015). Defluorination removes all toxicity. This defluorinating enzyme was first purified and characterised in 1965 and named fluoroacetate dehalogenase (Goldman, 1965). Most recently, a completely different enzyme fold, the haloacid dehalogenase superfamily, has also been shown to contain enzymes capable of defluorinating fluoroacetate (Chan et al., 2022).

Earlier tests of certain fluoroacetate dehalogenases with α,α‐difluorocarboxylic acids showed negligible activity, suggesting that their catalytic framework, evolved for the natural product fluoroacetate, was insufficient to react with perfluorinated carbon centers (Alexandrino et al., 2018; Goldman, 1965; Kurihara et al., 2003; Liu et al., 1998; Walker & Lien, 1981). Moreover, thiolytic displacement of fluoride from fluoroacetate was shown to be catalysed by a class of mammalian glutathione transferases, but they were inactive with difluoroacetate (Board & Anders, 2005). It is well known that increasing fluorine substitution on an sp3 carbon increases the bond strength of each bond compared to monofluorinated carbon atoms (Lemal, 2004). The C–F bond dissociation energy for difluoroacetate is enhanced by 5.8 kcal/mol over monofluoroacetate, explaining the greater resistance of the former to enzymatic attack (Yue et al., 2021).

Now, naturally‐evolved enzymes catalysing the enzymatic removal of both fluorine atoms of difluoroacetate have been identified, despite the more difficult displacement reaction they have to perform and that difluoroacetate is not known to be a natural product. Moreover, three enzymes show comparable rates with difluoroacetate as with fluoroacetate (Khusnutdinova et al., 2023), suggestive that the stronger C–F bonds of the perfluorinated carbon do not impose a rate limitation. The three enzymes are homologues, but differ significantly in sequence identity and are found in diverse genera: *Dechloromonas*, *Nostoc*, and *Polaromonas*. Given that a significant number of fluoroacetate defluorinating enzymes have been discovered (Chan et al., 2011; Donnelly & Murphy, 2009; Goldman, 1965; Kurihara et al., 2003), it seems likely that, over time, others will be found to be reactive with perfluorinated alkyl carbon atoms. Despite the greater catalytic power of enzymes that hydrolytically cleave both bonds of a difluoromethylene carbon, the active sites of the didefluorinases and monodefluorinases are highly similar. The mechanistic explanation for the extra catalytic power of the recently described enzymes to cleave the –CF_2_‐carbon‐to‐fluorine bonds is presently unknown, but can be revealed over time.

More recently, perfluorinated alkenoic acids were shown to undergo reductive defluorination catalysed by an electron bifurcating flavin‐iron–sulfur enzyme system (Yu et al., 2023). Electron bifurcation enzymes separate electron pairs into single electrons with high and low redox potentials, respectively, and achieve the lowest potentials known to biology (Buckel & Thauer, 2018; Müller et al., 2018). The reduction of perfluorinated acids was catalysed by CarCDE (Yu et al., 2023), and this enzyme system is produced by multiple *Acetobacterium* spp, a widespread genus found in anaerobic environments. CarCDE has been demonstrated to participate in the reduction of the lignin breakdown product caffeic acid, which is linked to ATP production, and its X‐ray structure has been solved (Demmer et al., 2018; Hess et al., 2013). Yu et al. (2023) presented evidence that perfluorinated alkenoic acids react with CarCDE and undergo defluorination. Computational docking showed a precise fit of *E*‐perfluoro‐4‐methylpent‐2‐enoic acid, (CF_3_)_2_CFCF=CFCOOH, in a reactive configuration positioned at the CarC enzyme flavin cofactor that participates directly in the reduction. Homologues to the *Acetobacterium* CarC protein are found in wastewater treatment plants globally, suggesting that this promiscuous defluorinating activity is widespread in nature. Another ATP‐dependent low‐potential substrate‐reducing enzyme system has been shown to catalyse defluorination from an aromatic ring (Tiedt et al., 2016). Most recently, many new electron‐bifurcating enzyme systems have been discovered and shown to attain a reduction potential as low as −911 mV vs the standard hydrogen electrode (Alleman & Peters, 2023; Mostafa et al., 2022; Wise et al., 2022). These recent reports offer an as‐yet‐underexplored potential for PFAS biodegradation.

Another recent study directly demonstrated a surprising degradative vulnerability of perfluorocarboxylic acids, which are a major PFAS class in widespread use and tightly regulated in many nations (Thomas et al., 2023). Perfluorooctanoic acid (PFOA), and shorter chain perfluorocarboxylic acids, was shown to undergo complete defluorination using mild reagents and temperatures, even 40°C (Trang et al., 2022). This discovery showed that a mild base in aprotic solvents catalyses PFOA decarboxylation and generates a very reactive carbanion intermediate, which spontaneously undergoes a series of energetically favourable defluorination reactions, even at low temperatures. Most microbes contain decarboxylases and the evolution of an enzymatic counterpart can be easily envisioned. A recent study showed biodegradation of fluorinated carboxylic acids and proposed initiating decarboxylation reactions, although no enzymes were identified directly (Xiao et al., 2023).

This Opinion article does not deny the obvious: C–F bonds are among the strongest covalent bonds. However, the biological and chemical examples above, coupled with other reports of microbiological PFAS defluorination, suggest that C–F bonds in many commercial PFAS are susceptible to enzymatic attack (Huang & Jaffé, 2019; Jin et al., 2023; Xiao et al., 2023). In light of that, I argue below that the environmental persistence of PFAS is best explained by evolutionary insufficiency and not inherent enzyme inadequacy.

## PFAS ARE TRULY “FOREIGN” TO BIOLOGY

The biodegradation of most newly introduced anthropogenic chemicals has been observed to evolve rapidly due to: (1) many commercial chemicals resembling natural products (2) the great number of Prokaryotes on Earth, (3) rapid microbial division rates increasing chances of adaptive mutations, and (4) a propensity for horizontal gene transfer mixes adaptive mutations across genomes (Wackett & Robinson, 2020). Industrial chemicals are sometimes described as “xenobiotic,” or foreign to biology, but most anthropogenic chemicals resemble naturally occuring compounds. For example, polychlorinated biphenyls (PCBs) were at one time considered to be very unlike natural products and non‐biodegradable (Sundström et al., 1976). In 1988, PCBs were found to be biodegraded in anaerobic sediments by bacteria using them as their final electron acceptor to support their energy metabolism (Brown et al., 1988). Subsequently, thousands of chlorinated natural products were discovered, many of them aromatic compounds, and some structurally resembling PCBs (Gribble, 2015). Consistent with that, genes encoding reductive dehalogenases of the type reactive with PCBs were found to be prevalent in the genomes of certain marine bacteria that are proposed to rely on reductive dehalogenation for energy conservation (Fincker & Spormann, 2017). New knowledge showed that PCBs should not be considered foreign to biology.

This scenario that played out with PCBs is unlikely to repeat with PFAS. Despite intensive efforts to discover new natural products for pharmaceuticals, polyfluorinated compounds are not known to exist in nature, and monofluorinated compounds are very rare. The only enzyme currently characterised for catalysing the biological formation of C–F bonds is fluorinase (Dong et al., 2004). It uses the inherent reactivity of the sulfonium cation of S‐adenosylmethionine to drive the formation of a C–F bond. This mechanism is not amenable to catalysing multiple fluorination reactions at the same carbon atom. In contrast to the limited slate of natural fluorinated compounds, humans have created more than 19 million fluorinated formulations (Barnabas et al., 2022), of which more that 7 million are PFAS (Schymanski et al., 2023). This tidal wave of new compounds are new to the Earth within the last century. Evolutionary adaption over 3.6 billion years of life on Earth occurred in the absence of PFAS. Now, PFAS biodegradation requires the simultaneous creation of multiple enzymatic and transport functions, as discussed in the next section.

## MULTIPLE EVOLUTIONARY ADAPTIONS ARE REQUIRED FOR PFAS BIODEGRADATION, WITH LIMITED BENEFITS FOR THE ORGANISM

**FIGURE 1:**
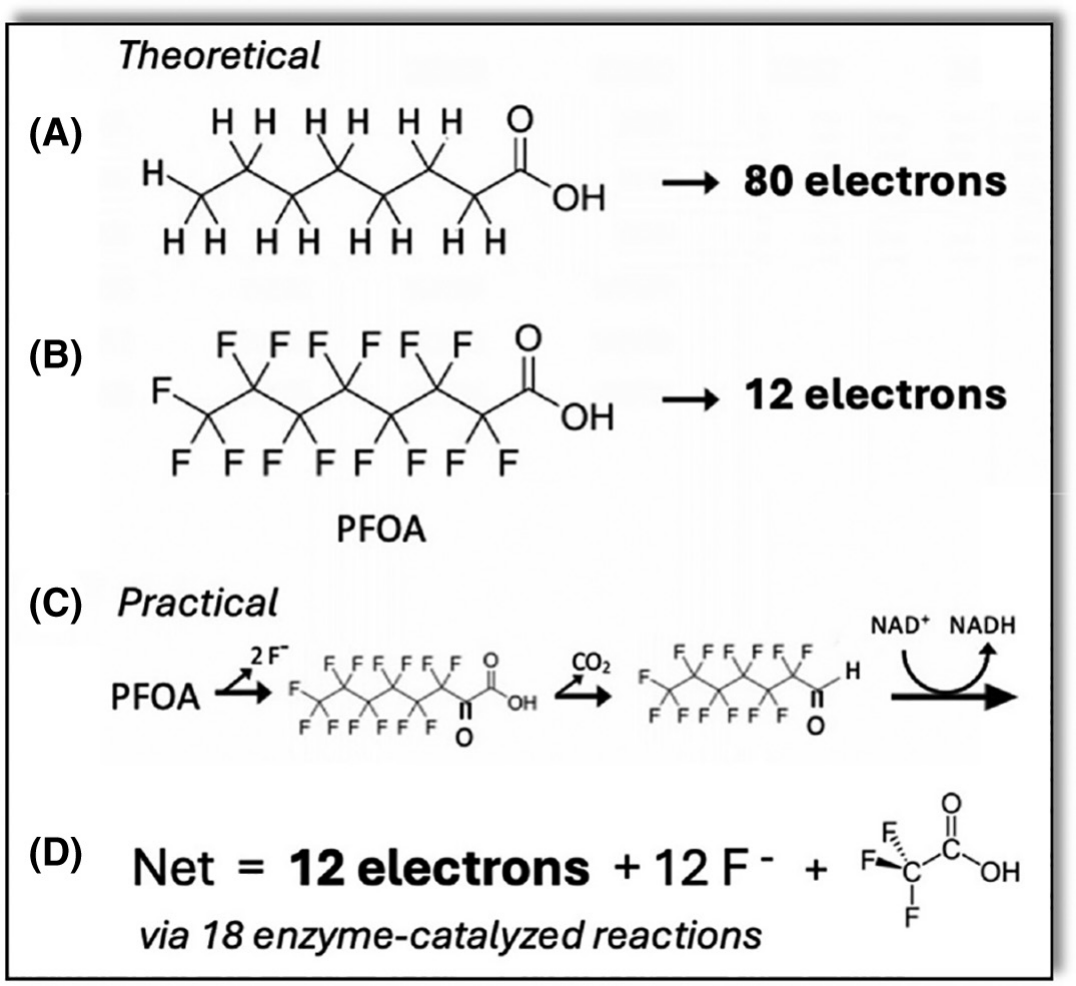
Comparison of metabolism of octanoic acid and a theoretical metabolism of perfluorooctanoic acid (PFOA). (A) Octanoic acid oxidation via β‐oxidation and TCA‐cycle oxidation of four acetyl‐CoA. (B) Hypothetical oxidation of PFOA yielding 12 electrons. (C) Hypothetical pathway of PFOA via hydrolytic defluorination, α‐decarboxylation, and oxidation carried out iteratively. (D) Compilation of the PFOA pathway that stops with release of 12 fluoride anions and trifluoroacetic acid (TFA). TFA is more recalcitrant to defluorination and likely would remain untransformed.

Evolution must bring together multiple biological functions simultaneously to efficiently degrade heavily fluorinated compounds. For evolutionary selection to occur, microbes must: (1) transfer PFAS across the membrane (known defluorinating enzymes are in the cytoplasm), (2) express the requisite enzymes, (3) carry out multiple C–F bond cleavage reactions by a cohort of enzymes, and (4) use the subsequent non‐fluorinated metabolites to produce ATP and new cell material. Evolution is driven by selective pressure. Figure 1 compares the selective advantage of metabolising fatty acids and the same chain length perfluorinated acids. The mechanism must be completely different as the deprotonation step in the former has no counterpart as F+ cannot be displaced. A theoretical metabolic pathway using a defluorinase, decarboxylase and dehydrogenase iteratively (Figure 1C) is biochemically plausible, although not known to exist currently. This might require multiple enzymes acting on different chain lengths. Even if the genes encoding such a pathway were to combine into one organism, it is important to consider that the energy yield would be far lower than that obtained for the β‐oxidation of fatty acids. The main point here is that the carbon atoms are highly oxidised, the only energy available is in the C‐C bonds and that is difficult to be realised.

Natural evolution occurs in stages, and in fits and starts (Jacob, 1977), and this is also true for evolutionary adaption to PFAS. For example, metabolising compounds with reasonable energy content connected to a perfluoro group of interest would be a reasonable initial step. Indeed, PFAS telomers that contain several methylene carbons connected to a perfluoroalkyl chain are known to be biodegradable by a number of microorganisms, suggesting that nature has already adopted this approach through natural evolution (Dinglasan et al., 2004; Kim et al., 2014; Shaw et al., 2019). Microbial oxidation of the non‐fluorinated methylene carbons provides energy to support growth. This gives microbes a foothold for the evolution of subsequent telomer defluorination.

## FLUORIDE TOXICITY IMPOSES A STRONG NEGATIVE SELECTION AGAINST PFAS BIODEGRADATION

In addition to the low energy content of highly oxidised PFAS, their metabolism will produce copious levels of fluoride anion. Fluoride is known to be highly toxic to all cellular life (Baker et al., 2012). Fluoride minerals are abundant in the Earth's crust, but microbes have, with very rare exceptions, not incorporated fluorine intracellularly. A study testing the total elemental composition of three dozen diverse bacteria did not detect any fluorine, despite the sensitivity of their methods that allowed them to detect very low traces of tin, barium and lead (Novoselov et al., 2013). This is readily explainable from an extensive literature survey which demonstrated that microbes go to great lengths to export intracellular fluoride due to its strong toxicity (Ji et al., 2014; McIlwain et al., 2021). As little as 0.1 mM fluoride has been shown to inhibit essential enzymes that contain magnesium, calcium, and other metals (Marquis et al., 2003; Qin et al., 2006).

Enzymatic organofluorine defluorination by cytoplasmic enzymes releases fluoride intracellularly and that has recently been shown to strongly inhibit growth and decrease cell viability. Dodge et al. (2024) engineered *Pseudomonas putida* ATCC12633 to express a fluoroacetate dehalogenase that was reactive with α‐fluorophenylacetic acid and 2‐fluoropropionic, producing mandelic acid and lactic acid, respectively, to support growth. A very high level of fluoride stress was observed. A higher degree of fluoride stress was manifested with 2‐fluoropropionic acid due to the lower energy content, leading to a loss of cell viability. Negative impacts of enzymatic defluorination were observed although *P. putida* KT2440 previously showed relatively high resilience to fluoride supplementation in growth media (Calero et al., 2022). Moreover, most bacteria are less tolerant to fluoride than *Pseudomonas* spp.

Another recent study also illustrated the importance of fluoride toxicity management in supporting microbial PFAS defluorination. In Yu et al. (2023), partial defluorination of a perfluoroalkenoic acid was demonstrated to only occur in cells containing the defluorinating enzymes AND a functioning fluoride export system. One bacterium had the defluorinating enzymes but did not catalyse defluorination in vivo. It was shown to have a deletion in part of the *crcB* gene encoding a fluoride exporter. This supports the idea that defluorinating enzymes exist but are insufficient. They must be paired with robust fluoride stress management functions for a bacterium to survive the biodegradation of fluorinated compounds. In both of the studies described above, only one fluoride anion per molecule was released. The release of 15 fluoride anions from perfluorooctanoic acid would be expected to impose an unmanageable fluoride stress level for most, or perhaps all, naturally occurring prokaryotes.

## MOST DOCUMENTED CASES OF NEWLY EVOLVED BIODEGRADATION OCCUR VIA POSITIVE SELECTION

**FIGURE 2:**
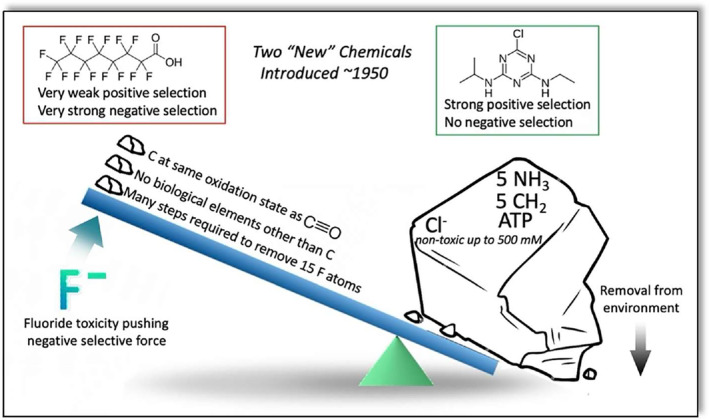
Comparison of the “weight” of selective pressure for atrazine, a herbicide, at the top right and perfluorooctanoic acid (PFOA) at the top left. Atrazine metabolism yields 5 nitrogen atoms for ammonia, 5 reduced carbon atoms to generate cell metabolites and ATP. Chloride anion is non‐toxic. PFOA contains carbon at the oxidation state of carbon monoxide, no other nutrient atoms, and would require many steps to remove 15 fluoride anions, and fluoride anion is highly toxic to cells.

Biodegradation occurs most robustly in nature when it is driven by selective pressure. For example, for decades following its introduction, the herbicide atrazine was biodegraded slowly in soils such that remaining atrazine injured susceptible crops the following year (Frank, 1966; Frank & Sirons, 1985; Kaufman & Kearney, 1970). Several decades later, the half‐life of atrazine in soils globally had shortened appreciably, reaching a half‐life of several days in some soils (Shaner & Henry, 2007; Shapir et al., 2007). After atrazine started degrading more rapidly in global soils, bacteria from six continents were isolated with the atrazine metabolising genes *atzABC*. The *atzABC* genes were nearly identical in sequence, and typically found on plasmids (De Souza, Seffernick, et al., 1998; Krutz, Shaner, Weaver, et al., 2010; Krutz, Shaner, & Zablotowicz, 2010). Most of the bacteria, or consortia of bacteria (De Souza, Newcombe, et al., 1998), were able to grow on atrazine as a sole source of nitrogen, a scarce resource in many soils. Atrazine contains five nitrogen atoms, none of the products were toxic, and the alkyl functional groups contained carbon at an average oxidation level that provides a strong selective pressure (Figure 2). The major evolutionary adaption was a series of point mutations in a deaminase gene to give rise to a novel dechlorinase enzyme, AtzA, that initiated the metabolism of atrazine (Seffernick et al., 2001). Additionally, multiple genes had to assemble, *atzABCDEF*, to make a complete metabolic pathway to liberate all of the growth supporting metabolites, ammonia and alkylamines (Shapir et al., 2007).

Biodegradation of PFAS have a much steeper evolutionary slope to climb for the reasons given above. Perfluorooctanoic acid (PFOA) is shown as an example to illustrate the multiple evolutionary impediments for PFAS biodegradation (Figure 2).

## CONCLUSIONS

Albert Einstein said, “We cannot solve our problems with the same thinking we used when we created them.” PFAS were created by industry largely to be chemically persistent in applications such as heat transfer, phase separation, and long‐term pest control. These widely known chemical properties understandably gave rise to the perception that microbial enzymes will prove catalytically inadequate to cleave C–F bonds other than those in fluoroacetate and a few other more monofluorinated compounds. As Einstein suggested, we should move away from our original idea and combine forces to solve the PFAS problems we have created.

To summarize, this Opinion argues that PFAS are (1) increasingly shown to be susceptible to enzymatic defluorination, (2) inherently foreign to biology and will require many new metabolic combinations to biodegrade, (3) necessarily dependent on robust fluoride toxicity abatement mechanisms for biodegradation to proceed, and (4) biodegradable only when cellular energy systems are present that can withstand and support fluoride stress management.

## FUNDING INFORMATION

No funding information provided.

## CONFLICT OF INTEREST STATEMENT

The author declares no conflict of interest for this article.

## Data Availability

The data that support the findings of this study are available from the corresponding author upon reasonable request.
